# P-1099. Correlation Analysis of Cefiderocol *In vitro* Activity and *In vivo* Efficacy Against *Acinetobacter baumannii* Strains with Difficult-to-Read MIC Endpoints

**DOI:** 10.1093/ofid/ofae631.1287

**Published:** 2025-01-29

**Authors:** Hidenori Yamashiro, Motoyasu Onishi, Naomi Anan, Boudewijn L DeJonge, Christopher M Longshaw, Miki Takemura, Yoshinori Yamano

**Affiliations:** Shionogi & Co., Ltd., Toyonaka, Osaka, Japan; Shionogi & Co., Ltd., Toyonaka, Osaka, Japan; Shionogi & Co., Ltd., Toyonaka, Osaka, Japan; Shionogi Inc., Florham Park, New Jersey; Shionogi B.V., London, England, United Kingdom; Shionogi & Co., Ltd, Toyonaka, Osaka, Japan; Shionogi & Co., Ltd., Toyonaka, Osaka, Japan

## Abstract

**Background:**

Cefiderocol (FDC) is a siderophore-conjugated cephalosporin with broad activity against Gram-negative bacteria, including *Acinetobacter baumannii*. For some isolates, determining the minimum inhibitory concentration (MIC) endpoints for FDC can be difficult due to the trailing growth phenomenon. Recently guidance on how to determine the MIC when trailing occurs has been updated in the CLSI M100 document, to enhance MIC reproducibility. In this study, we determined MICs of *A. baumannii* strains that show difficult to determine MIC endpoint due to trailing according to the updated CLSI guidance and analyzed correlations between MICs and *in vivo* efficacy.

In vivo bacterial growth or reduction from start of treatment with cefiderocol against Acinetobacter baumannii in the neutropenic murine thigh infection model using humanized pharmacokinetic exposure of cefiderocol.

**Figure:**
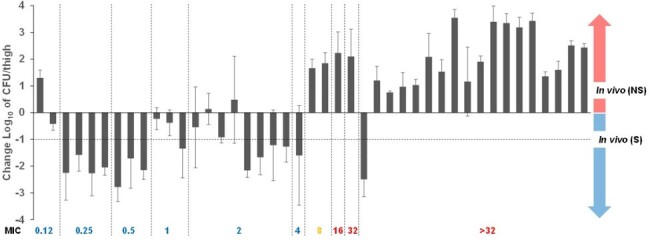

In vivo (S): mean of reduction in bacterial cell number at 24 hours after initial treatment with cefiderocol (mean of change <0 log10 colony forming unit (CFU) /thigh) CFU/thigh), in vivo (R): mean of increase in bacterial cell number at 24 hours after initial treatment with cefiderocol (mean of change ≥0 log10 CFU/thigh). MIC values shown blue, orange, and red indicate susceptible, intermediate, and resistant, respectively, according to CLSI guideline 2024.

**Methods:**

MICs were determined according to the 2024 CLSI guidance using iron-depleted cation-adjusted Mueller-Hinton broth (ID-CAMHB) from BD-BBL for 43 *A. baumannii* isolates, including 13 strains that show severe trailing. All strains had been evaluated *in vivo* in the murine thigh infection model using humanized pharmacokinetic exposure of FDC (1).

Categorical agreements between MICs and in vivo efficacy in murine thigh infection model

**Figure:**
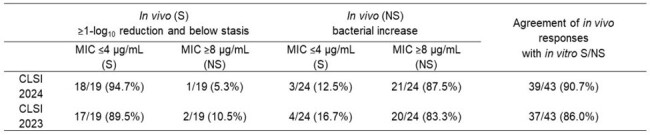

Susceptible (S), Non-susceptible (NS), breakpoints as published by CLSI (2024), In vivo (S): mean of reduction in bacterial cell number at 24 hours after initial treatment with cefiderocol (mean of change <0 log10 colony forming unit (CFU)/thigh, in vivo (R): mean of increase in bacterial cell number at 24 hours after initial treatment with cefiderocol (mean of change ≥0 log10 CFU/thigh).

**Results:**

FDC MIC range of tested *A. baumannii* strains was 0.12 to >32 μg/mL. Among 19 strains that showed a reduction in bacterial load after treatment with FDC in the murine thigh infection model, 94.7% (18/19 strains) were categorized as FDC susceptible (Figure and Table 1). Among 24 strains that showed increase in bacterial load after treatment with FDC *in vivo*, 87.5% (21/24 strains) were categorized as FDC non-susceptible. The categorical agreement of MICs and *in vivo* efficacy was 90.7% (39/43). As for the 13 strains that showed severe trailing, the categorical agreement of MICs and *in vivo* efficacy was 84.6% (11/13, Table 2). The updated CLSI guidance did not impact the overall agreement that was obtained earlier using the previous CLSI guidance (Table 1, 2).

Categorical agreements between MICs and in vivo efficacy in murine thigh infection model for 13 strains that showed severe trailing

**Figure:**
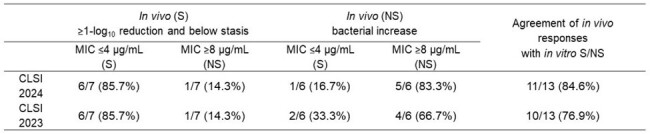

Susceptible (S), Non-susceptible (NS), breakpoints as published by CLSI (2024), In vivo (S): mean of reduction in bacterial cell number at 24 hours after initial treatment with cefiderocol (mean of change <0 log10 colony forming unit (CFU)/thigh, in vivo (R): mean of increase in bacterial cell number at 24 hours after initial treatment with cefiderocol (mean of change ≥0 log10 CFU/thigh).

**Conclusion:**

Our correlation analysis between MICs and *in vivo* efficacy in thigh infection model confirms the validity of MIC endpoint determinations described in CLSI M100 document, including strains that show severe trailing.

**Reference:**

1. Monogue L, *et al. Antimicrob Agents Chemother*. 2017;61(11):e01022-17.

**Disclosures:**

**Hidenori Yamashiro**, Shionogi & Co., Ltd.: Employee **Motoyasu Onishi, PhD**, Shionogi & Co., Ltd.: Employee **Naomi Anan, MSc**, Shionogi & Co., Ltd.: Employee **Boudewijn L. DeJonge, PhD**, Shionogi Inc.: Employee **Christopher M. Longshaw, PhD**, Shionogi BV: Employee **Miki Takemura, n/a**, Shionogi & Co., Ltd.: Employee **Yoshinori Yamano, PhD**, Shionogi & Co., Ltd.: Employee

